# Supplementary material for: Hamster Sperm Possess Functional Na+/Ca2+-Exchanger 1: Its Implication in Hyperactivation
Source: Int J Mol Sci. 2023 May 17;24(10):8905. doi: 10.3390/ijms24108905 (PMC10219178; doi:10.3390/ijms24108905)
Supplement: Supplementary file 1 [file ijms-24-08905-s001.zip › Figure S1.pdf]

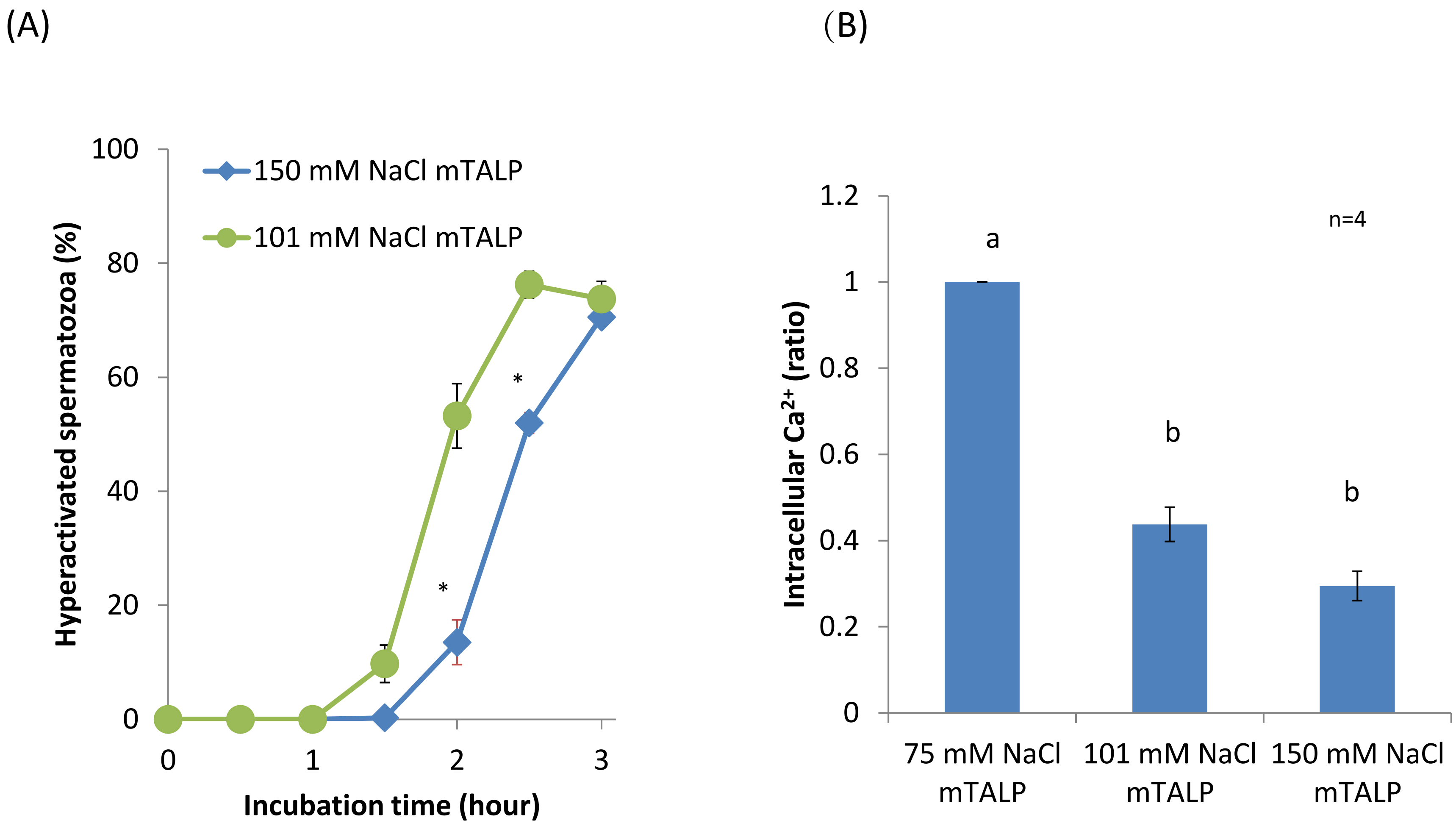

Supplementary Figure S1: NCX-like activity is involved in the regulation of hamster sperm hyperactivation. The effect of extracellular Na+ on hyperactivation (A) and intracellular Ca2+ levels (B) were investigated. * or different superscripts indicate significant differences among them.
