# Supplementary material for: Hamster Sperm Possess Functional Na+/Ca2+-Exchanger 1: Its Implication in Hyperactivation
Source: Int J Mol Sci. 2023 May 17;24(10):8905. doi: 10.3390/ijms24108905 (PMC10219178; doi:10.3390/ijms24108905)
Supplement: Supplementary file 1 [file ijms-24-08905-s001.zip › Figure S2.pdf]

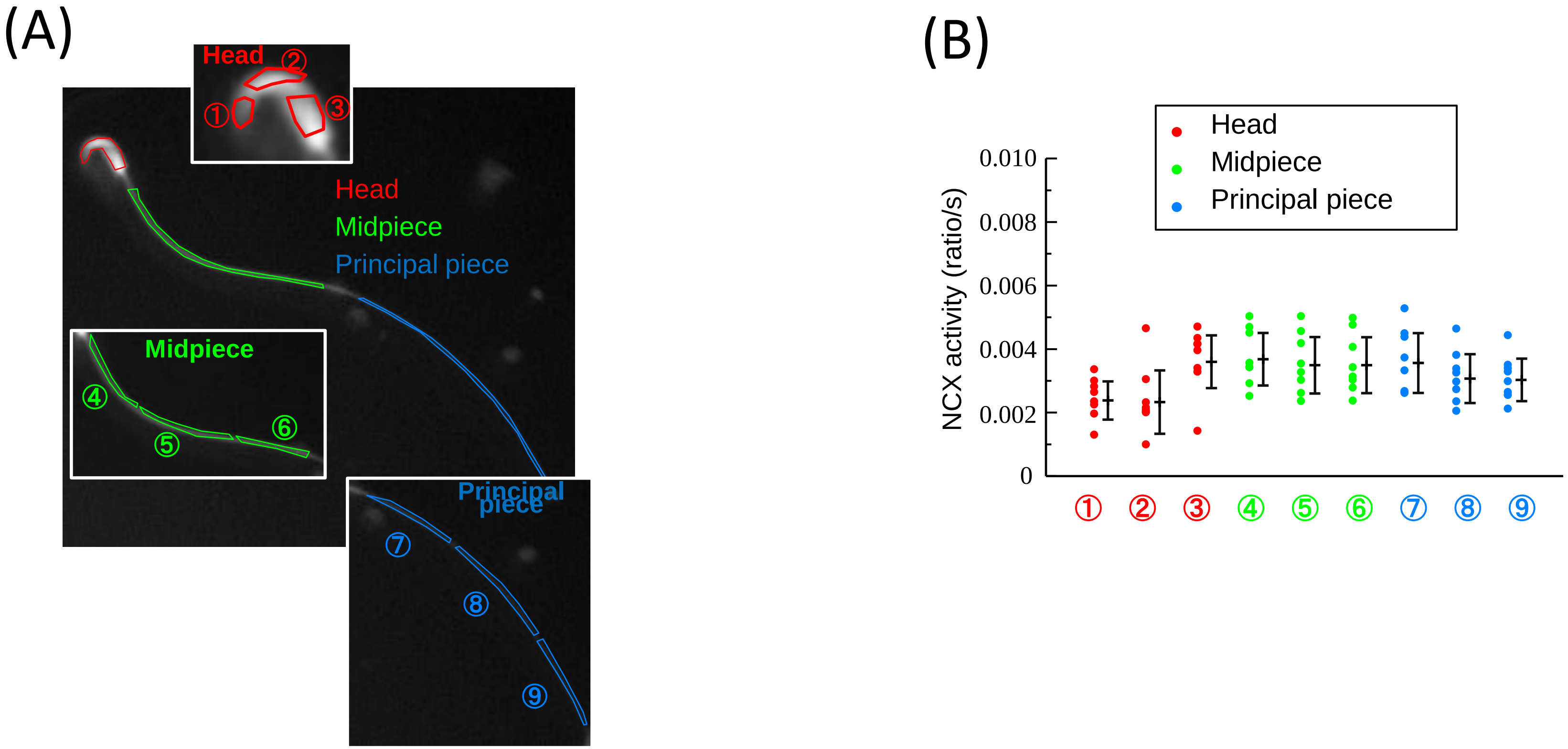

Supplementary Figure S2:The detailed region-specific measurement of NCX activity in hamster. The ROIs for de-tailed measurement (A) and the region-dependent NCX activities were shown (B)
