# Supplementary material for: Hamster Sperm Possess Functional Na+/Ca2+-Exchanger 1: Its Implication in Hyperactivation
Source: Int J Mol Sci. 2023 May 17;24(10):8905. doi: 10.3390/ijms24108905 (PMC10219178; doi:10.3390/ijms24108905)
Supplement: Supplementary file 1 [file ijms-24-08905-s001.zip › Figure S3.pdf]

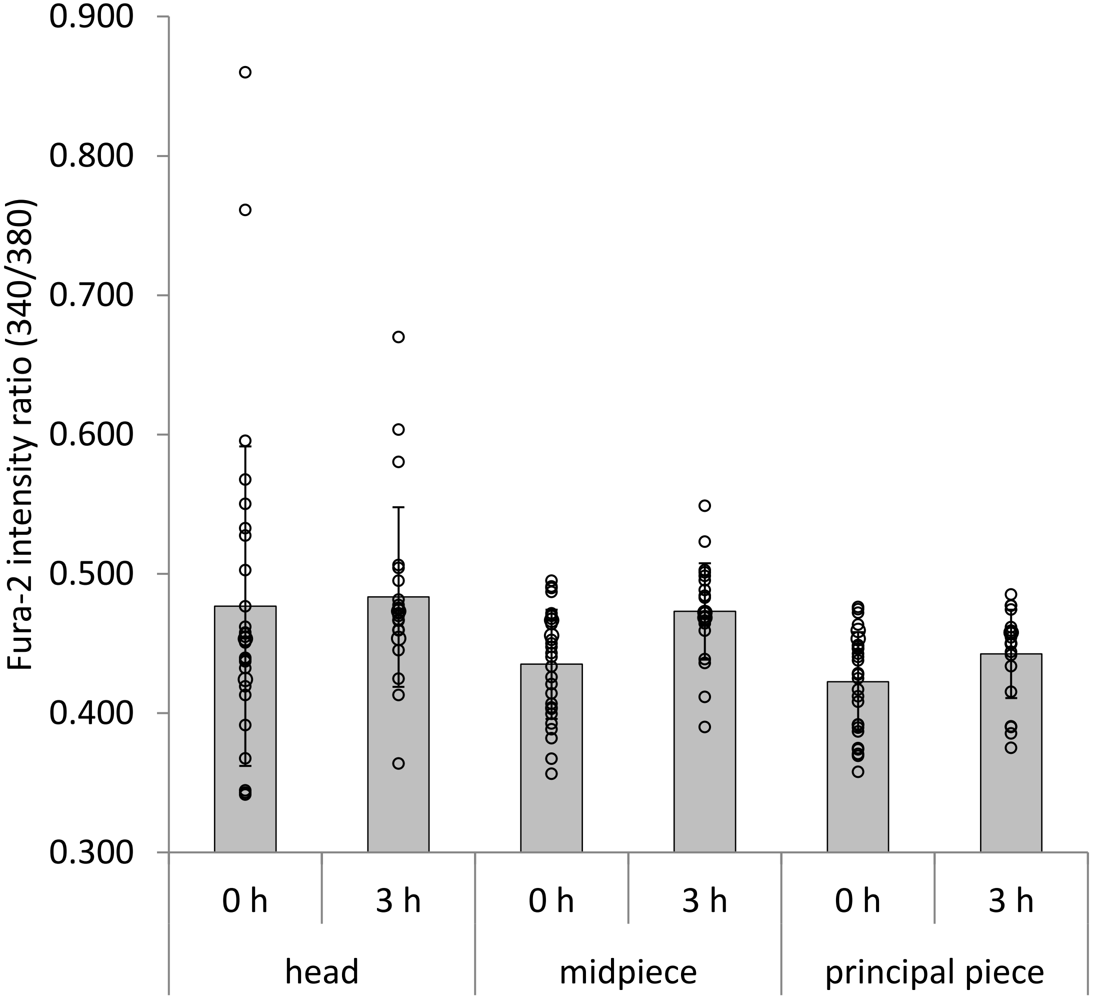

	head		midpiece		principal piece	
	0 h	3 h	0 h	3 h	0 h	3 h
	0.568	0.670	0.495	0.549	0.472	0.462
	0.595	0.467	0.490	0.488	0.448	0.459
	0.462	0.481	0.407	0.495	0.371	0.454
	0.432	0.445	0.392	0.469	0.358	0.477
	0.343	0.364	0.367	0.390	0.389	0.390
	0.437	0.504	0.404	0.464	0.387	0.415
	0.341	0.413	0.388	0.411	0.429	0.385
	0.860	0.495	0.491	0.523	0.463	0.477
	0.503	0.506	0.403	0.459	0.374	0.442
	0.761	0.459	0.447	0.466	0.417	0.450
	0.533	0.474	0.487	0.472	0.446	0.457
	0.440	0.460	0.472	0.464	0.474	0.450
	0.453	0.473	0.467	0.469	0.453	0.458
	0.476	0.478	0.464	0.472	0.440	0.444
	0.457	0.475	0.450	0.501	0.425	0.450
	0.458	0.425	0.470	0.503	0.476	0.485
	0.419	0.466	0.414	0.499	0.408	0.449
	0.455	0.603	0.433	0.436	0.392	0.390
	0.451	0.580	0.468	0.439	0.442	0.375
	0.438	0.472	0.443	0.484	0.449	0.474
	0.424	0.454	0.456	0.472	0.459	0.458
	0.527	0.470	0.453	0.483	0.438	0.434
	0.451		0.421		0.446	
	0.550		0.426		0.412	
	0.345		0.356		0.392	
	0.367		0.382		0.369	
	0.391		0.399		0.375	
	0.413		0.440		0.428	
mean	0.477	0.483	0.435	0.473	0.423	0.443
SD	0.115	0.064	0.039	0.035	0.036	0.032

Figure S3 : Enlarged Fig. 4B and raw data of Fura-2 intensity ratio
